# Mechanisms of Cardiac Inflammation in Heart Failure: Role of Dietary Patterns, Nutrients, and Therapeutic Strategies

**DOI:** 10.3390/nu18061005

**Published:** 2026-03-22

**Authors:** Andreas Mavroudis, Christos Fragoulis, Kyriaki Mavromoustakou, Panagiotis Iliakis, Konstantinos Tsioufis, Christina Chrysohoou

**Affiliations:** 1 First Cardiology Clinic, School of Medicine, University of Athens, 11527 Athens, Greece; christosfragoulis@yahoo.com (C.F.); mavromoustakoukiriaki@yahoo.gr (K.M.); panayiotisiliakis@gmail.com (P.I.); ktsioufis@gmail.com (K.T.); 2 Department of Medicine, Division of Cardiology, Angiology and Internal Emergency Medicine, Knappschaft Kliniken University Hospital Bochum, Ruhr University Bochum, 44892 Bochum, Germany

**Keywords:** cardiac inflammation, pathophysiology, inflammatory cytokines, diet effect, therapeutic intervention

## Abstract

**Background:** Systemic inflammation is a key driver of heart failure (HF) progression across all ejection fraction (EF) phenotypes, with diet emerging as a modifiable factor influencing cardiac metabolism and inflammatory signaling. This narrative review integrates current evidence on the inflammatory mechanisms underlying HF, their links with common comorbidities and emerging anti-inflammatory therapeutic strategies, with a particular focus on the role of nutrition in supporting healthy cardiac metabolism. **Methods:** We searched MEDLINE/PubMed, EMBASE, Web of Science, the Cochrane Library, Scopus and reference lists of relevant publications using terms related to systemic inflammation, dietary patterns and HF prioritizing high-impact studies on nutrition–inflammation–HF interactions published from 2000 onward. **Results:** Major HF comorbidities sustain chronic, low-grade inflammation through elevated cytokine activity. Dietary patterns—especially those with high Dietary Inflammatory Index (DII)—substantially shape inflammatory milieu. The Mediterranean diet appears to have a favorable inflammatory profile with reduction in circulating pro-inflammatory biomarkers, especially *C*-reactive protein (CRP) and interleukin-6 (IL-6). Established therapies for HF with reduced ejection fraction and vagus nerve stimulation elicit anti-inflammatory efficacy through cytokine suppression. Sodium glucose cotransporter-2 (SGLT2) inhibitors demonstrate positive metabolic effects and anti-inflammatory actions through decrease in IL-6 and tumor necrosis factor-α (TNF-α). Interleukin-1 blockade has produced heterogeneous clinical outcomes, while definitive findings examining the role of IL-6 inhibitors in inflammation suppression and possible benefit on cardiac outcomes are anticipated. Preliminary data show the potential synergistic effects of dietary patterns/nutrients and pharmacological agents combination on improvement of endothelial function and attenuation of the fibrotic process, although there is a need for further research in large-scale trials. **Conclusions:** Systemic inflammation demonstrates a key role in HF initiation and progression, and the effect of diet on inflammatory pathways is central. Dietary patterns targeting inflammation-related mechanisms (inflammasome, gut dysbiosis) can lead to attenuation of systemic inflammatory response and restoration of cardiac metabolic flexibility. A deeper mechanistic discernment of cardiac inflammatory cascades, together with identification of HF subpopulations with excessive inflammatory activity, may facilitate the design of targeted randomized controlled trials (RCTs) aiming for novel personalized, inflammation-targeted HF therapies with potential clinical benefit.

## 1. Introduction

HF affects more than 60 million people globally [1]. Systemic inflammation represents a common pathobiological hallmark of both acute and chronic HF as it drives 30–50% progression via metabolic dysregulation [2]. Accumulating evidence indicates a broad spectrum of pathophysiological mechanisms contributing to HF initiation and progression [3]. Comorbidities involved in the pathogenesis of HF are implicated in cardiac and systemic inflammation [4]. Emerging evidence suggests that dietary patterns can modulate cardiac metabolism via inflammasome inhibition and subsequent decrease in inflammatory biomarkers, mitochondrial reactive oxygen species (ROS) reduction and changes in gut microbiota [2,5,6,7]. Established therapies that are given in heart failure with reduced ejection fraction (HFrEF) show potential anti-inflammatory effects through inflammatory biomarkers suppression [3]. Cardiac resynchronization therapy (CRT) and its effect on suppression of inflammation is accompanied by conflicting results, while vagus nerve stimulation has shown promising results regarding the suppression of inflammatory cytokines; however, whether this translates into a reduction in cardiac events incidence remains unclear [8]. This review aims to provide key preclinical, clinical, and epidemiological evidence on the role of systemic inflammation in HF. The review focuses on underlying pathophysiological mechanisms, inflammatory links with common HF comorbidities, emerging anti-inflammatory therapeutic strategies and potential synergies of combinations of dietary patterns or specific nutrients with pharmacological agents, emphasizing the need for identification of HF subpopulations with excessive inflammatory activity and targeted attenuation of inflammation via personalized dietary patterns and adjunctive therapies.

## 2. Materials and Methods

### 2.1. Search Strategy

A structured literature search was conducted using terms related to systemic inflammation and HF, including “systemic inflammation in heart failure,” “cardiac inflammatory pathophysiology,” “HF comorbidities and inflammation,” “anti-inflammatory therapies in HF,” and “diet, nutrition, and cardiac inflammation.” Searches were performed across MEDLINE/PubMed, EMBASE, Web of Science, the Cochrane Library, Scopus, and reference lists of relevant publications. Keywords encompassed combinations of “heart failure,” “systemic inflammation,” “pathophysiology,” inflammatory cytokines, comorbid conditions, dietary patterns, nutrients and anti-inflammatory interventions using Boolean operators (AND/OR). Given the broad mechanistic and clinical scope of this topic, we used a targeted narrative review approach rather than a systematic review framework. The search strategy prioritized recent, high-impact studies focused on RCTs/meta-analyses, with relevance to nutrition–inflammation interactions [e.g., DII], rather than aiming for exhaustive coverage. Only English-language publications from 2000 onward were included, with the final search completed in January 2026.

### 2.2. Study Selection

Studies were selected based on their relevance to the role of systemic inflammation in HF pathophysiology—such as cytokine-mediated fibrosis, endothelial dysfunction, and mitochondrial oxidative stress—as well as its contribution to comorbidity-driven disease progression [e.g., renin–angiotensin–aldosterone system (RAAS) activation and gut dysbiosis]. Both pharmacological and non-pharmacological interventions, including nutritional approaches, were considered. Eligible publications included original research articles, RCTs, cohort studies, meta-analyses, and narrative or systematic reviews published in peer-reviewed journals. Priority was given to landmark clinical trials (e.g., CANTOS, PREDIMED), mechanistic studies published between 2020 and 2026, and nutrition-focused investigations such as those evaluating dietary inflammatory indices or Mediterranean diet effects on inflammatory biomarkers. Experimental therapies (e.g., ziltivekimab, elamipretide) and dietary interventions (e.g., berry consumption or omega-3 supplementation) were included when supported by robust mechanistic evidence. Studies were excluded if they lacked a clear HF focus, demonstrated methodological limitations, or addressed standard HF therapies without inflammatory or nutritional relevance. Non-English publications were not considered.

### 2.3. Data Extraction

Data extraction was independently performed by two reviewers (CF and AM) using standardized data collection forms. Extracted variables included study design and population characteristics, inflammatory mechanisms [e.g., Toll-like receptor 4 (TLR4), NOD-like receptor protein 3 (NLRP3) signaling], biomarkers [e.g., galectin-3, soluble suppression of tumorigenesis-2 factor (sST2)], inflammatory contributions of comorbidities, nutritional influences [e.g., effects of polyphenols on gut microbiota and Trimethylamine *N*-oxide (TMAO) production], intervention outcomes [e.g., reductions in high-sensitivity CRP (hs-CRP), HF-related events), safety considerations, and study limitations. Discrepancies were resolved through consensus or consultation with senior reviewers (CC and KT). This approach enabled a balanced synthesis of clinically relevant evidence supporting diet- and nutrient-based strategies to attenuate inflammation in HF, in line with Nutrients’ emphasis on metabolic health.

## 3. Results

### 3.1. Pathophysiology of Cardiac Inflammation

Several pathophysiological mechanisms are involved in the development of cardiac inflammation, which is prevalent in both acute and chronic HF and among all HF categories [3].

Biomarker patterns vary across the EF spectrum in HF [3]. Patients with HFrEF mainly display a “cardiac stretch” signature, characterized by markers linked to cellular proliferation and metabolism [3]. In contrast, heart failure with preserved ejection fraction (HFpEF) is dominated by biomarkers of cardiac inflammation and shows a broader, more heterogeneous biomarker profile, mirroring the clinical diversity of these patients [3]. This may be explained by the greater burden of comorbidities in HFpEF patients, such as diabetes mellitus, arterial hypertension, chronic kidney disease and obesity [4]. Heart failure with mildly reduced ejection fraction (HFmrEF) occupies an intermediate position, with abnormal levels of both stretch-related and inflammatory biomarkers. These observations suggest that although some core mechanisms are shared across EF categories, inflammation may contribute more specifically and strongly at one end of the spectrum. The inflammatory/pro-fibrotic paradigm is one such mechanism proposed to underlie HFpEF pathogenesis [3].

### 3.2. HF Comorbidities and Inflammation

Diabetes mellitus, arterial hypertension, chronic kidney disease (CKD) and obesity are common comorbidities in HF patients leading to systemic inflammation and having unfavorable effects on the kidneys, the muscles and the pulmonary system through sodium and water retention, worsening sarcopenia and causing an increase in vascular resistance [4]. Hyperglycemia in diabetes mellitus triggers chronic inflammation and enhances oxidative stress, which in turn contributes to the production of stress kinases leading to insulin resistance along with activation of inflammatory cytokines and production of ROS [9,10]. In patients with arterial hypertension, increased activity of RAAS and Angiotensin II contribute to oxidative stress and vascular inflammation by promoting the expression of nicotinamide adenine dinucleotide phosphate (NADPH) oxidases and endothelin-1, leading to the production of ROS and enhancement of oxidative stress [11]. CKD is characterized by a sustained pro-inflammatory state [12]. As kidney function declines, circulating inflammatory cytokines are increased through both elevated expression and declined renal elimination, along with increased expression of IL-1α in circulating monocytes, leading to an elevated risk of cardiovascular disease [12]. Inflammation in CKD is also driven by oxidative stress through uremia, activation of RAAS and Angiotensin II [12]. Moreover, elevated carbamylated low-density lipoprotein (LDL), a posttranslational modified form of LDL, has been documented in CKD patients exerting its inflammatory action through endothelial dysfunction and impaired endothelial nitric oxide (NO) bioavailability [12]. Obesity, with both visceral and epicardial adipose tissue expansion, is also implicated in HF, especially HFpEF [12,13]. Activation of hypoxia-inducible factor 1α (HIF-1α) along with accumulation of pro-inflammatory long-chain fatty acids result in infiltration and activation of inflammatory cells within the visceral adipose tissue with subsequent expression of inflammatory cytokines, systemic and myocardial inflammation [12]. The expansion of epicardial adipose tissue, through promotion of leptin, TNF-α, IL-1β and IL-6 expression and concurrent reduction in adiponectin release, is independently associated with all-cause mortality and HF hospitalizations in HFpEF subpopulations [12].

### 3.3. Endothelial Dysfunction and Inflammation

Endothelial inflammation of microvasculature leads to the production of adhesion molecules with subsequent entrapment of leucocytes, myofibroblast formation and collagen deposition [4]. Endothelial dysfunction also promotes the production of ROS, impairing the availability of NO and resulting in cardiomyocyte hypertrophy [4]. Additionally, TLR4, a pattern-recognition receptor (PRR), is predominantly expressed in the heart and plays a significant role in the pathogenesis of inflammation in HF promoting the expression of interleukins and adhesion molecules, mainly IL-1β, IL-6 and intercellular adhesion molecule (ICAM-1) [4].

### 3.4. The Role of Biomarkers in Cardiac Inflammation

#### 3.4.1. Pro-Inflammatory Cytokines

Pro-inflammatory cytokines are implicated in myocardial dysfunction and adverse remodeling. They mainly exert a negative inotropic effect and contribute to increased myocardial stiffness through promotion of cardiac fibrosis [14].

CRP is synthesized in the liver in response to IL-6 signaling and reflects systemic inflammatory status [3]. In subjects free of ischemic heart disease or HF and after adjustment of traditional risk factors, elevated baseline CRP levels conferred a higher risk of HF events. Similar outcomes of greater cardiovascular, all-cause mortality and hospitalization risk were found in chronic HF patients [3].

TNF-α is mainly produced by the immune system cells and to a lesser extent by cardiomyocytes [15]. It exerts a negative inotropic effect and activates the expression of ICAM-1 and vascular cell adhesion molecule (VCAM-1) leading to the recruitment and entrapment of neutrophils in the microcirculation and causing endothelial dysfunction [14]. Increased circulating levels of TNF-α have been observed in patients with HF or cardiomyopathy and are related with both cardiac systolic and diastolic dysfunction and increased mortality in both HFpEF and HFrEF subtypes [14]. Its effects are mediated through binding toTNF receptors 1 (TNFR1) and 2 (TNFR2). Through its binding with TNFR1, TNF-α drives cardiomyocyte death and fibrosis, while its binding with TNFR2 facilitates infarct scar formation and promotes angiogenesis and repair in chronic HF setting [16,17,18].

IL-1β, as an inflammatory cytokine, impairs endothelial function, contributes to extracellular matrix (ECM) remodeling and provokes the accumulation of neutrophils in inflammatory tissues leading to adverse cardiac remodeling [14]. IL-6 exerts a negative inotropic effect mostly in HFpEF patients and contributes to myocardial cell hypertrophy [3,14]. Evidence shows that IL-8 and IL-18 are associated with adverse cardiovascular events, whereas IL-33 has anti-inflammatory properties, preventing Angiotensin II-induced cardiac fibrosis [3].

Lipopolysaccharide (LPS), a bacterial endotoxin, stimulates the secretion of IL-1 and TNF [19]. LPS is increased in patients with acute decompensated heart failure (ADHF), as the intestinal edema in congestive HF leads to translocation of bacteria and LPS from the gut to circulation [19].

#### 3.4.2. Fibrosis-Associated Biomarkers

Transforming growth factor-β (TGF-β) is related with endothelial dysfunction, promotion of myocardial stiffness, HF manifestation and cardiac fibrosis [20].

C-C Motif Chemokine Ligand 2 (CCL2), also known as Monocyte Chemoattractant Protein-1 (MCP-1), is an inflammatory molecule involved in the activation of leukocytes, cardiac systolic dysfunction and fibrosis and increased mortality, especially in advanced HF patients [14].

Galectin-3, a chimera-type, glycan-binding protein, influences inflammatory and apoptotic procedures and, thus, is associated with adverse cardiac remodeling and the fibrotic process [3,21]. Galectin-3 contributes to the activation and migration of neutrophils at sites of inflammation and activation of TGF-β with subsequent activation of fibroblasts and formation of fibrotic tissue [21]. Increased levels of galectin-3 have been confirmed in cardiomyopathies, heart failure and adverse cardiovascular events, including acute myocardial infarction, as it contributes to atheroma formation and progression [21].

sST2 is a marker of fibrosis that is released in response to vascular congestion and pro-inflammatory triggers [3,20]. Higher concentrations of sST2 are linked with cardiovascular mortality and HF events both in HFpEF and HFrEF phenotypes [3,20].

#### 3.4.3. Damage-Associated Molecular Patterns (DAMPs)

Uric acid serves as both a marker of systemic inflammation and an adverse prognostic indicator in HF [22]. Through its binding to TLR2/TLR4 receptors and activation of NLRP3 inflammasome, uric acid triggers the production of IL-1β and TNF-α and immune cell recruitment [22]. Uric acid also drives oxidative stress by upregulating xanthine oxidase, boosting ROS production and promoting cardiac hypertrophy and fibrosis [3].

In cardiac inflammation, mitochondria seem to play a significant role with the production of ROS and mitochondria DAMPs (MitoDAMPs), leading to secretion of pro-inflammatory cytokines, myocardial dysfunction and adverse remodeling [23].

S100A8/A9, also known as calprotectin, is a heterodimeric complex of calcium-binding proteins—members of the alarmin family—that is released from monocytes and neutrophils upon inflammatory stimuli [24]. S100A8/A9 interacts with TLR4 leading to nuclear factor κΒ (NF-kB) activation and expression of pro-inflammatory cytokines, such as TNF-α, IL-6 and IL-17 [24]. In addition, S100A8/A9 leads to neutrophil extracellular trap (NET) formation, resulting in neutrophil activation, adhesion and chemotaxis and subsequent release of proteases and coagulation factors, which contribute to development and progression of atherosclerosis [24]. S100A8/A9 also interacts with the vascular cells promoting endothelial dysfunction, atherosclerotic plaque progression and potential plaque rupture showing its crucial role in acute coronary syndrome (ACS) [24]. Patients with ACS demonstrate increased levels of S100A8/A9, with higher concentrations being associated with worse cardiovascular outcomes [24]. S100A8/A9 also plays a key mechanistic role in ischemic HF pathogenesis via several pathways [24]. Following cardiac injury, this alarmin interacts with TLR4 and the Receptor for Advanced Glycation Endproducts (RAGE) leading to NF-kB-driven oxidative stress [24]. S100A8/A9 also promotes cardiomyocyte apoptosis by inducing mitochondrial permeability transition pore (mPTP) opening and calcium dysregulation along with inflammatory cell recruitment and activation of fibroblasts, fueling a persistent inflammatory cycle, myocardial fibrosis and ventricular dysfunction [24].

#### 3.4.4. Congestion-Related Biomarkers and the Role of Neutrophils

Natriuretic peptides (NP), including B-type natriuretic peptide (BNP) and *N*-terminal pro BNP (NT-proBNP) are neurohumoral biomarkers, with ventricular volume or pressure contributing to their secretion [3]. However, inflammation is also an independent trigger of NP release [3,20]. Evidence shows an independent positive correlation of IL-6 with NT-proBNP [3,14]. In healthy individuals, the administration of LPS led to a marked increase in NT-proBNP levels [3,20]. NP mediate much of this inflammation–HF association by reflecting subclinical ventricular dysfunction triggered by inflammation; however, their utility in monitoring inflammation in clinical practice remains unclear [3,20].

Neutrophils acting by release of inflammatory cytokines and by their accumulation in inflammatory areas can lead to cardiac tissue remodeling [3]. The neutrophil-to-leucocyte ratio (NLR) strongly predicts HF mortality, major cardiovascular events, hospitalizations and chronic kidney disease in the elderly [3]. Baseline NLR has been independently associated with all-cause mortality and HF hospitalization regardless of EF and has been correlated with IL-6, sST2 and NT-proBNP [3]. Despite neutrophil’s clear link to HF severity and mortality across different HF etiologies, targeted therapies remain scarce [3].

Table 1 summarizes pro-inflammatory, fibrosis-associated biomarkers and DAMPs, their mechanism of action and impact on cardiac inflammation/HF.

**Figure 1:**
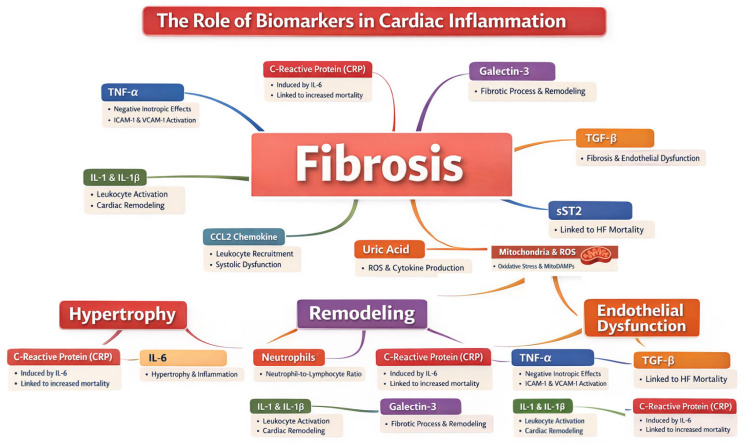
Biomarkers, DAMPs mechanism of action and effect on cardiac inflammation. Abbreviations: CRP: *C*-reactive protein, IL: interleukin, TNF-α: tumor necrosis factor-α, ICAM-1: intercellular adhesion molecule, VCAM-1: vascular cell adhesion molecule, CCL2: C-C Motif Chemokine Ligand 2, ROS: reactive oxygen species, RAAS: renin–angiotensin–aldosterone system, GDF-15: growth/differentiation factor 15, MitoDAMPS: mitochondrial damage-associated molecular patterns, TGF-β: transformin growth factor-β, sST2: soluble suppression of tumorigenesis-2 factor.

Figure 1 summarizes the main inflammatory, fibrosis-associated biomarkers and DAMPs, their mechanism of action, and their effect on cardiac inflammation.

### 3.5. Molecular Pathways Involved in Cardiac Metabolism

The heart’s metabolism is highly adaptive with fatty acid oxidation being the main source of myocardial Adenosine Triphosphate (ATP), while glucose, lactate and ketone bodies contribute to cardiac contractility [25]. Metabolic flexibility, the ability to shift among fuel sources, is crucial for optimal cardiac performance [25]. Diets rich in saturated fats or high-glycemic carbohydrates lead to downregulation of 5′ Adenosine monophosphate (AMP)-activated protein kinase (AMPK), a sensor for low energy states promoting glucose uptake and fatty acid oxidation, and sirtuin 1/peroxisome proliferator-activated receptor gamma coactivator 1-alpha (SIRT1/PGC1-α), a regulator of mitochondrial biogenesis [25]. Additionally, the excessive consumption of saturated fats and simple sugars causes dysregulation of nuclear factor kappa-light-chain-enhancer of activated B cells (NF-κB) and mitogen-activated protein kinase (MAPK) pathways with subsequent accumulation of ROS, stimulation of inflammatory biomarkers and promotion of the fibrotic process [25]. On the other hand, balanced dietary patterns, e.g., Mediterranean diet, promote AMPK, SIRT1/PGC1-α pathways along with peroxisome proliferator-activated receptor alpha (PPARα), a molecular regulator of fatty acid β-oxidation, leading to efficient mitochondrial function, improved cardiac metabolism and reduction in oxidative stress [25].

### 3.6. Effect of Dietary Patterns/Nutrients in Cardiac and Systemic Inflammation

Previous studies have shown a direct link of diet with both cardiac and systemic inflammation [26]. The DII is a validated scoring system that incorporates 45 dietary components weighted according to their association with inflammatory biomarkers (IL1-β, IL-6, IL-8, TNF-α, CRP, homocysteine) based on scientific evidence from a literature review of ~1000 articles [26,27]. DII values range from 7.98 to −8.87, with higher scores indicating a more pro-inflammatory diet and lower values a more-anti-inflammatory diet [27]. Its calculation is implemented via various dietary assessment tools, most commonly food frequency questionnaires [26]. An inflammatory dietary pattern, reflected by higher DII, has been associated with an increased risk of cardiovascular disease and mortality and an elevated incidence of inflammation-associated diseases, such as cancer and neurocognitive disorders [26]. Higher levels of DII are also linked with elevated LDL and an increased risk of hypertension [26].

#### 3.6.1. Mediterranean Diet

The traditional Mediterranean diet represents a predominantly plant-based dietary pattern characterized by a high intake of seasonal vegetables and fruits, with extra virgin olive oil being the main source of dietary fat [5]. Fermented dairy products, mainly yogurt and feta cheese, are consumed regularly, while fish is consumed 2–3 times weekly [5]. White meat is preferred over consumption of red and processed meat, water is the principal beverage, while wine is moderately consumed and almost exclusively in meals [5]. A Mediterranean diet, via promotion of SIRT/PGC1-α and upregulation of Nuclear factor erythroid 2-related factor 2 (Nrf2) molecular pathways [25], seems to have a more favorable inflammatory profile, with significant reduction in both oxidative stress biomarkers, such as F2-isoprostane, and circulating pro-inflammatory biomarkers, including CRP, IL-6 and fibrinogen [5]. In adults aged <60 years with cardiovascular disease, a Mediterranean diet significantly reduced IL-6 levels by up to 20% [6]. It also has a favorable impact on circulating levels of platelet-activating factor (PAF) and lipoprotein-associated phospholipase A2 (Lp-PLA2), both involved in the initiation and progression of inflammation and atherosclerosis [7]. Additionally, it contributes to deceleration of atherosclerosis progression with a reduction in carotid intima-media thickness and carotid plaque height [5].

The role of gut microbiota in the initiation and progression of inflammation is central, as alterations in gut microbiota composition, commonly referred to as dysbiosis, are known to be associated with low-grade chronic inflammation [5]. High-fat diets are linked with increased expression of TMAO, derived from dysbiotic gut microbiota, resulting in endothelial dysfunction and promotion of fibrosis [25]. Long-term adherence to Mediterranean diet, reduces TMAO and enhances beneficial gut bacteria versus harmful bacteria taxa [5]. A Mediterranean diet rich in fiber promotes the production of short-chain fatty acids by gut bacteria, leading to improved mitochondrial function, suppression of the inflammation process and reduction in oxidative stress [25]. Adherence to this dietary pattern is associated with a decreased incidence of cardiovascular events, stroke and neurocognitive disorders, through reduction in oxidative stress and the anti-inflammatory properties of macro- and micronutrients—including polyphenols [5]. Both observational and interventional studies indicate that the anti-inflammatory impact of a Mediterranean diet is also extended to patients with immune-mediated inflammatory diseases, e.g., inflammatory bowel syndrome, rheumatoid arthritis and psoriasis [5]. In patients with metabolic syndrome, commonly characterized by increased inflammatory burden, a Mediterranean diet without weight loss significantly reduced plasma CRP [28]. Finally, in patients with coronary heart disease, the DII was significantly improved after a 6-month adherence to a Mediterranean diet [5].

In patients after acute myocardial infarction, adherence to antioxidant-rich diet, in addition to physical activity into a structured cardiac rehabilitation program, favorably influences inflammatory biomarkers [29]. In people at risk of cardiovascular disease, compliance with a healthy diet enriched with either carbohydrates, protein or unsaturated fat and low in cholesterol and sodium can lead to reduction in both cardiac injury and inflammatory biomarkers, mitigating subclinical cardiac damage and inflammation [30].

#### 3.6.2. Polyphenols and Flavonoid-Rich Diet

Polyphenols and unsaturated fats found in the Mediterranean diet increase the expression of microRNAs (miRNA) involved in cardiac contractility, with an average daily amount of 30–50 g olive oil, 200–300 g vegetables and 20–40 g nuts being beneficial for improvement of cardiac contractility and protection of cardiomyocytes against the fibrotic process [25]. Berries are a great source of polyphenols and antioxidant nutrients, e.g., vitamin C, E and beta-carotene and exert their protective effect against inflammation and cardiometabolic disorders [31]. Berry consumption is also associated with reduction in total cholesterol, LDL and triglycerides along with fasting glucose and insulin and inflammatory biomarkers, e.g., CRP, that is primarily attributed to the flavonoids, especially anthocyanins, with their antioxidant properties [31]. Flavonoids exert their antioxidant and anti-inflammatory properties through mitigation of ROS and NF-κB pathway with subsequent reduction in pro-inflammatory cytokines [32,33]. Additionally, flavonoids act through inhibition of phospholipase A2, cyclooxygenase and lipoxygenase interfering in the metabolism of arachidonic acid and resulting in reduced expression of prostaglandins, leukotrienes and thromboxanes, while through inhibition of TGF-β, flavonoids attenuate cardiac fibrosis [32,33]. In patients with non-alcoholic fatty liver disease (NAFLD), the polyphenol naringenin reduces serum total cholesterol and triglycerides, while catechin reduces Alanine Aminotransferase (ALT) and Aspartate Aminotransferase (AST), and increases high-density lipoprotein (HDL) [34]. In NAFLD patients, quercetin, commonly found in fruits and vegetables, inhibits the TNF-α-mediated inflammatory pathway by impeding MAPK or enhancing peroxisome proliferator-activated receptor gamma (PPAR-γ) activity [34].

#### 3.6.3. Ketogenic and Low-Carbohydrate Dietary Patterns

Regarding a ketogenic diet, its anti-inflammatory effects are expressed through: (a) the ketone bodies, the main energy substrate produced by fat oxidation, (b) the substantial reduction or elimination of simple sugars, (c) the reduction in total carbohydrate intake, and (d) the anti-inflammatory properties of omega-3-fatty acids [35]. B-hydroxybutyrate, the main ketone body, exerts its anti-inflammatory effect through inhibition of NLRP3 inflammasome, which is involved in the production and release of pro-inflammatory cytokines [35]. Low carbohydrate intake, as part of a ketogenic diet, leads to a reduction in pro-inflammatory markers and omega-3 fatty acids exert their anti-inflammatory action through displacement of arachidonic acid in membrane phospholipids and subsequent reduction in inflammatory eicosanoids [35]. These anti-inflammatory effects indicate that a ketogenic diet could serve as a potential non-pharmacological intervention in heart failure and cardiovascular diseases [35].

#### 3.6.4. Vegetarian, Vegan and Gluten-Free Diet

Vegetarian and vegan diets—i.e., excluding meat and all animal products, respectively—represent intensified plant-based patterns [25]. Randomized trials and meta-analyses link them to lowered total cholesterol, LDL-C, apolipoprotein B, HbA1c, and body weight, especially in overweight/obese individuals, type 2 diabetes patients, or those with CV risk [25]. These diets combine low saturated fat/cholesterol with high fiber, complex carbs, unsaturated fats, and polyphenol-rich plants enhancing insulin sensitivity and metabolic flexibility [25]. At the cardiac level, these diets promote efficient mitochondrial oxidation, the enhancement of AMPK-SIRT1-PGC-1α activity and attenuation of NF-κB inflammatory pathway—mitigating oxidative stress and remodeling [25].

Evidence shows that adherence to a gluten-free diet is linked with significant reduction in CRP, indicating the potential attenuation of systemic inflammatory response following gluten abstinence [36]. Similar results have been shown with the adherence to dietary patterns rich in high-quality carbohydrates from whole-grain bread and oats and lower total fat, mainly saturated fatty acids [37].

#### 3.6.5. Coenzyme Q10, Probiotics and Selenium

Coenzyme Q10 (CoQ10) plays a critical role in mitochondrial ATP production, reduction in ROS and oxidative stress and improvement of endothelial function [38]. CoQ10 supplementation at a dose range of 200–300 mg/day for at least 12 weeks significantly attenuates circulating levels of CRP, IL-6 and TNF-α [39]. However, there was no significant effect on NT-proBNP levels and left ventricular ejection fraction (LVEF) [1]. Despite its limitations, Q-SYMBIO trial demonstrated a significant reduction in the 2-year all-cause mortality, cardiovascular death and HF hospitalizations with CoQ10 supplementation at 300 mg/day as adjunctive therapy in patients with chronic HF compared with placebo [38]. However, larger trials are required to validate these findings [38].

The consumption of 300 mL of probiotic yogurt showed a significant reduction in oxidated LDL compared to controls and an increase in soluble tumor necrosis factor-like weak inducer of apoptosis (sTWEAK) [1]. Decreased levels of this factor are associated with metabolic syndrome, atherosclerosis and endothelial dysfunction [40].

Selenium deficiency has been associated with exercise intolerance in HF patients, whereas in animal studies high selenium diet attenuated oxidative stress and fibrosis, making this micronutrient a potential supportive approach among the available therapeutic options in HF patients [41].

Despite the aforementioned data, RCTs are required to determine whether diets with a low inflammatory profile can lead to meaningful improvement in health outcomes [42].

**Figure 2:**
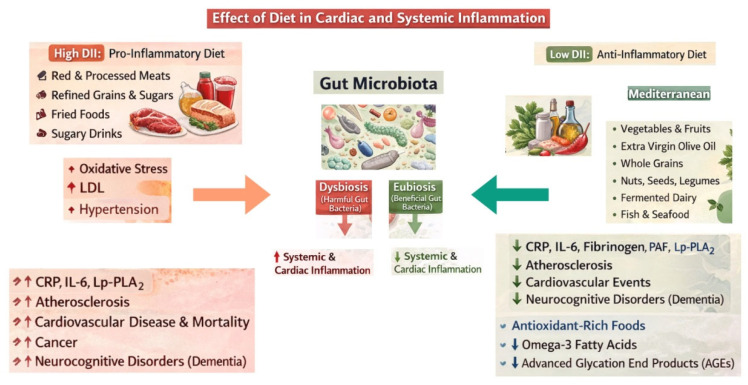
Effect of diet on cardiac and systemic inflammation. Abbreviations: DII: dietary inflammatory index, LDL: low-density lipoprotein, CRP: *C*-reactive protein, IL: interleukin, Lp-PLA_2_: Lipoprotein Phospholipase A_2_, PAF: platelet-activating factor.

Figure 2 summarizes the main effects of diet in cardiac and systemic inflammation.

Table 2 summarizes the effects of dietary patterns/nutrients on inflammation/metabolism and involved molecular pathways.

### 3.7. Therapy

#### 3.7.1. Cornerstone HFrEF Therapy and Its Potential Anti-Inflammatory Role

The established therapy in patients with HFrEF has been studied regarding its potential anti-inflammatory properties. Small, single-center studies have presented a modest reduction in inflammatory cytokines by carvedilol and bisoprolol; however, it is unknown if this is associated with reduction in adverse cardiac events [3].

Angiotensin-converting enzyme inhibitors (ACEI) and angiotensin receptor blockers (ARB) exert their anti-inflammatory properties through blockade of angiotensin II, resulting in decrease in CRP, NP and IL-6 and positive cardiovascular benefits, especially in HFrEF patients [3]. Clinical studies showed the anti-inflammatory effect of sacubitril/valsartan, an angiotensin receptor neprilysin inhibitor (ARNI), through reduction in both CRP and sST2; however, this effect seems to be inferior compared with ACEi and ARB [3].

Mineralocorticoid receptor antagonists (MRA) reduce the expression of both pro-inflammatory cytokines (TNF-α, IL-6, IFN-γ) and fibrosis markers (Galectin-3, sST2) resulting in reduction in oxidative stress and the fibrotic process [3].

SGLT2 inhibitors have emerged as a cornerstone therapy across HFrEF and HFpEF phenotypes [45]. Their pleiotropic effects regarding cardioprotection and renoprotection also involve modulation of inflammatory cascades leading to a decrease in IL-6, TNF-α and interferon gamma (IFN-γ) [45]. SGLT2 inhibitors also have a favorable metabolic profile reversing the release of adipokines and reducing adiponectin by the adipose tissue [3].

#### 3.7.2. Anti-Inflammatory Properties of Statins, *N*-3 Polyunsaturated Fatty Acids (*n*-3 PUFA) and Loop Diuretics

Statins are known to have anti-inflammatory and antioxidant effects, through blockade of TLR pathway, reduction in NF-κB signaling and decrease in CRP, ICAM-1, VCAM-1 [4,46].

*N*-3 polyunsaturated fatty acids (*n*-3 PUFA) are associated with improvement of left ventricular diastolic function and reduction in BNP in patients with chronic stable HF [43]. In a small-scale study *n*-3 PUFA improved LVEF accompanied by a reduction in both inflammatory cytokines (hs-CRP) and markers of fibrosis (sST2) [44].

Loop diuretics have also been linked with reduction in TNF-α, IL-1b, IL-6, IL-8 and IL-10, although the clinical significance is unclear [3].

#### 3.7.3. Colchicine, IL Inhibitors, TNF-Blocking Agents and Methotrexate

Colchicine decreases CRP and IL-6, inhibits secretion of IL-1 by neutrophils; however, its role in heart failure remains to be determined [3,4].

Regarding IL inhibitors, canakinumab—an IL-1β antagonist—reduced the composite of cardiovascular mortality and hospitalizations in patients with previous myocardial infarction and a hs-CRP ≥2 mg/L in CANTOS trial [3].

Anakinra, an IL-1 inhibitor, improved peak oxygen consumption (VO2max) in patients with recently decompensated HF [1]; however, it failed to shorten hospital stay duration or reduce HF hospitalization rates [4].

Ziltivekimab, a monoclonal antibody targeting IL-6, is currently under investigation in patients with subclinical inflammation [3,47,48]. HERMES trial is evaluating its efficacy in patients with HFmrEF or HFpEF and elevated NT-proBNP levels, while ZEUS trial examines the outcomes with the use of ziltivekimab versus placebo in patients with established atherosclerotic disease and CKD with an estimated glomerular filtration rate (eGFR) 15–60 mL/min/1.73 m^2^ [3,47,48].

Ustekinumab, a monoclonal antibody that inhibits IL-12 and IL-23, improved global longitudinal strain (GLS) and coronary flow reserve in patients with psoriasis and LVEF > 50%, without a history of primary cardiomyopathy, myocardial infarction or active myocardial ischemia [2,49]. However, in patients with high—but not low—baseline cardiovascular risk, ustekinumab significantly increased cardiovascular events [50].

Trials examining the potential benefit of TNF-blocking agents in inflammation and HF have been led to its discontinuation due to excessive mortality and morbidity in the treatment arm [19]. In the ATTACH trial infliximab increased all-cause mortality or HF hospitalization, while in the RENEWAL trial, the risk of HF hospitalization increased with etanercept [4,51].

CIRT trial demonstrated neutral effects of methotrexate, with no reduction in cardiovascular events in patients with stable atherosclerotic cardiovascular disease [4].

#### 3.7.4. Serelaxin, Cardiac Mitotropes and Nicotinamide Riboside

Serelaxin, a recombinant human relaxin-2 hormone and potent vasodilator, significantly reduced HF events in hospitalized patients with ADHF, although without impact on cardiovascular mortality [20].

Cardiac mitotropes enhance myocardial contractility through optimization of myocardial energetics [20]. In small studies, perhexilline showed positive metabolic effects through improvement of myocardial ATP synthesis [20]. Clinical evaluation in chronic HF revealed improvement of left ventricular function and symptom burden [20]. Trimetazidine prevents oxidation of fatty acids in mitochondria leading to modification of cardiac metabolism and subsequent improvement of left ventricular function. Elamipretide protects the mitochondrial membrane and attenuates the production of ROS in preclinical models [20]. However, RCTs are needed to further elucidate the potential positive effects of these agents [20].

Nicotinamide riboside, as a precursor of nicotinamide adenine dinucleotide (NAD), reduces the secretion of ROS, IL-1β, TNF-α and IL-6 acting as a potential and promising cardioprotective agent in both animal and human studies [23].

#### 3.7.5. Different Modes of Exercise and Potential Anti-Inflammatory Properties

Regarding the potential anti-inflammatory role of exercise, meta-analyses have shown a positive effect on the reduction in inflammatory biomarkers, especially TNF-α, IL-6 and hs-CRP [52]. A significant decrease in TNF-α was demonstrated in HFrEF patients performing high-intensity exercise, while reduction in IL-6 was seen in HFpEF patients, following aerobic exercise at high or intermediate intensity [52]. This exercise pattern also resulted in a significant reduction in hs-CRP across HFrEF and HFmrEF phenotypes [52].

#### 3.7.6. Interventional Approaches and Potential Anti-Inflammatory Effects on HF Setting

Numerous small-scale trials have tried to shed light on whether CRT attenuates the inflammatory milieu in HF patients; however, the results are rather conflicting [19]. Reductions in CRP, IL-6 and TNF has been reported following implantation of CRT, while other trials observed no significant changes in these biomarkers [19].

Vagus nerve stimulation of sensory afferent fibers triggers acetylcholine release into the reticuloendothelial system, suppressing pro-inflammatory cytokine production. In HFpEF patients, transcutaneous vagus nerve stimulation has been associated with improvement of GLS and quality of life—being a potential therapeutic approach in this target group [8].

Table 3 summarizes the main RCTs and meta-analyses evaluating the impact of pharmacological agents on inflammatory biomarkers and cardiovascular events.

#### 3.7.7. Combination of Diet/Pharmacological Agents-Potential Anti-Inflammatory Effects and Cardiovascular Benefits

Animal and small-scale observational human studies have shown that the combination of pharmacological agents with dietary patterns/nutrients may have a synergistic effect on cardiovascular health, promoting cardioprotection and reducing oxidative stress [25]. Combining a Mediterranean diet with statins notably reduces TMAO and pro-inflammatory cytokines, like IL-6, which results in improvement of endothelial function and ventricular compliance [25]. Metformin, when combined with a low-carbohydrate diet, enhances AMPK activity and mitochondrial function leading to improved exercise tolerance and reduction in oxidative stress [25]. The combination of a polyphenol-rich diet with ACE inhibitors attenuates inflammation, cardiac remodeling and the fibrotic process with an additional favorable effect on diastolic function [25]. Preliminary data from both human and animal studies have shown a possible favorable impact of Omega-3 PUFA-β-blockers combination on arrhythmia burden through promotion of PPARα, AMPK activities [25]. However, there is a need for well-designed trials, especially in HFpEF populations, where therapeutic options are markedly limited.

Table 4 summarizes nutrient–therapy synergies, the main involved molecular pathways and potential cardiovascular benefits.

## 4. Discussion

In this narrative review we examined the pathophysiological inflammatory pathways implicated in HF along all specific phenotypes, the effect of dietary patterns on cardiac inflammation and the impact of established therapies on down-regulation of the inflammatory process along with possible therapies with potential promising effects on cardiac and systemic inflammation in HF patients.

HFpEF displays a unique metabolic-inflammatory phenotype driven by comorbidities such as obesity, diabetes, and CKD and dominated by biomarkers of cardiac inflammation, whereas HFrEF is characterized by markers linked to cellular proliferation [3]. IL-6 exerts a negative inotropic effect and promotes myocardial hypertrophy [3,14]. Elevated IL-6 is associated with a higher incidence of diabetes and obesity, while HFpEF patients with increased levels of IL-6 demonstrate an impaired functional capacity with lower peak VO2 and higher NT-proBNP levels, indicating a greater cardiac congestion at rest [60].

Accumulating evidence demonstrates a close relationship between diet and inflammation, with a higher DII leading to a proportionate increase in cardiovascular disease and mortality along with elevated incidence of inflammation-mediated disorders [26]. Meta-analyses have demonstrated the anti-inflammatory profile of Mediterranean, polyphenol-rich, and omega-3-based dietary patterns, reducing both inflammatory and oxidative stress biomarkers [5,25,31,43,44]. However, trials particularly in HFpEF settings are limited.

Q-SYMBIO RCT showed a significant reduction in all-cause mortality and cardiovascular death with CoQ10 supplementation at 300 mg/day for 2 years compared with placebo in patients with chronic HF [38]. However, limitations of the trial regarding the 8-year enrollment period and the relatively small number of enrolled patients should prompt further caution in the interpretation of the results [38]. Future trials of CoQ10 with inclusion of HFpEF patients are necessary, given the limited therapeutic interventions in this setting [38].

Evidence shows that the ‘’Fantastic-Four’’ treatment of HF, especially HFrEF, has anti-inflammatory properties; however, it is still unknown if the decrease in inflammatory burden leads to clear clinical benefits and reduction in cardiovascular events [3]. RCTs examining the potential benefit of anti-IL-6 inhibitors in patients with systemic inflammation are still pending [3,47,48], while those examining TNF-α blocking agents have been discontinued due to increased risk of hospitalization, morbidity and mortality [4,19,51]. Although TNF-α drives cardiomyocyte death and fibrosis, its binding with TNFR2 facilitates infarct scar formation and promotes angiogenesis and repair in chronic HF [16,17,18]. TNF-α, via myocardial nitric oxide, can potentially lead to a reduction in β-adrenergic responsiveness, mitigating sympathetic overdrive toxicity [16,17]. These potential benefits undermine the traditional view of TNF-α as purely detrimental in chronic HF and can partially explain the failure of TNF-α blocking agents in HF settings [16,17].

Evidence from animal and small observational human studies suggests that combining dietary patterns or nutrients with pharmacological therapies may improve endothelial function, exercise tolerance and attenuation of the fibrotic process [25]. However, further research in well-structured, large-scale trials is needed. This concept is particularly relevant for HFpEF patients, who often demonstrate heightened inflammatory burden and limited treatment options [25].

This narrative review has several limitations inherent to its design and scope. First, as a targeted literature synthesis rather than a systematic review or meta-analysis, it may introduce selection bias despite structured searches across major databases (MEDLINE/PubMed, EMBASE, etc.) from 2000 to January 2026, potentially overlooking relevant non-English studies or lower-impact publications. Second, the heterogeneous nature of included studies—spanning preclinical models, small RCTs, observational cohorts, and mechanistic investigations—precludes quantitative pooling of effect sizes for dietary interventions (e.g., Mediterranean diet’s IL-6 reduction) or therapies (e.g., SGLT2 inhibitors on TNF-α), limiting generalizability across HF phenotypes (HFrEF, HFpEF, HFmrEF). Third, while prioritizing high-impact evidence, we focused on nutrition–inflammation interactions, without exhaustive coverage of genetic/epigenetic modifiers or long-term outcomes, and reliance on biomarker proxies (hsCRP, galectin-3) rather than hard endpoints (HF hospitalizations, mortality) which limit causal inferences. Finally, evolving data from ongoing trials (e.g., HERMES for ziltivekimab) were incorporated only up to the search cutoff, and real-world applicability of nutrient strategies (e.g., polyphenol dosing, adherence in geriatric HF) remains underexplored amid variability in gut microbiota responses.

## 5. Future Directions

Future directions should prioritize multicenter RCTs evaluating personalized anti-inflammatory interventions in high-risk HF subpopulations, such as those with elevated hs-CRP/IL-6 and metabolic dysregulation. Trials integrating Dietary Inflammatory Index scoring with wearables for dietary adherence could validate Mediterranean/polyphenol-rich patterns or ketogenic regimens as adjuncts to SGLT2 inhibitors/ARNI therapy, targeting endpoints like fibrosis imaging [cardiac magnetic resonance (CMR) T1 mapping}, HF events, and quality-of-life metrics. Mechanistic studies should elucidate nutrient synergies (e.g., ω-3 + selenium on NLRP3/gut-TMAO axis) via omics profiling (metabolomics, single-cell RNA-seq) to foster precision nutrition. High-IL-6 HFpEF cohorts warrant IL-6 inhibitors (ziltivekimab) combined with exercise protocols, while vagus stimulation merits larger outcome trials. Given the neutral results of trials with IL-1 inhibitors, future well-structured studies examining IL-1 blockade in patients with elevated baseline IL-1β or NLRP3 activity should be implemented. These approaches may help identify subgroups most likely to benefit from inflammation-targeted interventions and could establish scalable, lifestyle-first strategies for healthy cardiac metabolism, bridging *Nutrients*’ focus on diet/nutrients with personalized HF care.

## 6. Conclusions

Experimental and clinical evidence underscores the pivotal role of inflammation in both acute and chronic HF pathogenesis and across all ejection fraction phenotypes. Nutrition modulates inflammatory biomarkers and gut microbiota composition. While established HF therapies and selective anti-cytokine agents (IL-1 blockade) attenuate inflammatory burden, results remain mixed: CRT yields conflicting results regarding biomarker responses and trials with TNF-α inhibitors have been discontinued due to paradoxically worsened outcomes. These inconsistencies highlight the complexity of targeting inflammation in HF. In the era of personalized medicine, identification of HF subpopulations with excessive inflammatory activity along with targeting selective cytokine-driven pathways may serve optimal clinical benefit and resolve current controversies.

## Figures and Tables

**Table 1:** Biomarkers–mechanism of action/impact on HF.

Biomarker	Mechanism of Action/Impact on HF
CRP	Association with greater all-cause and cardiovascular mortality.
TNF-α	Negative inotropic effect, systolic and diastolic dysfunction, link with increased mortality.
IL-1	Endothelial dysfunction, adverse cardiac remodeling.
IL-6	Negative inotropic effect, link with myocardial hypertrophy.
IL-8, IL-18	Association with adverse cardiovascular events.
LPS	Stimulation of IL-1 and TNF, increased levels in ADHF.
TGF-β	Endothelial dysfunction, cardiac fibrosis.
CCL2	Systolic dysfunction, cardiac fibrosis.
Galectin-3	Atheroma formation and progression, adverse cardiac remodeling and fibrosis.
sST2	Promotion of cardiac fibrosis, link with increased cardiovascular mortality and HF events.
Uric acid	Link to cardiac fibrosis.
S100A8/A9	Stimulation of TNF-α, IL-6, IL-17, promotion of endothelial dysfunction, atherosclerosis progression, myocardial fibrosis, ventricular dysfunction.

Abbreviations: HF: heart failure, CRP: *C*-reactive protein, TNF-α: tumor necrosis factor-α, IL: interleukin, LPS: Lipopolysaccharide, ADHF: acute decompensated heart failure, TGF-β: transformin growth factor-β, CCL2: C-C Motif Chemokine Ligand 2, sST2: soluble suppression of tumorigenesis-2 factor.

**Table 2:** Effects of diet/nutrients on inflammation/metabolism and molecular pathways.

Diet/Nutrient (Reference)	Molecular Pathways	Effects on Inflammation/Metabolism
Mediterranean diet [5,6,25]	Activation of SIRT1/PGC1-α, Nrf2 upregulation	↓ IL-6 (20%), hs-CRP, ↑ gut diversity, ↓ TMAO improved endothelial function and lipid metabolism.
Berries (polyphenols) [31]	↓ NF-κB, phospholipase A2, cyclooxygenase and lipoxygenase inhibition, ↓ TGF-β	↓ CRP, ROS ↓ total cholesterol, fasting glucose, insulin levels ↓ cardiac fibrosis.
Keto diet/omega-3 fatty acids [35,43,44]	NLRP3 inhibition	↓ hs-CRP, sST2 eicosanoid shift.
Vegetarian and gluten-free diet [25,36]		↓ CRP
Coenzyme Q10 [39]		↓ CRP, IL-6, TNF-α
Probiotics [1]		↓ oxidated LDL, ↑ sTWEAK
Selenium [41]	↓ NF-κB	↓ oxidative stress, fibrosis.

Abbreviations: SIRT1/PGC1-α: sirtuin 1/peroxisome proliferator-activated receptor gamma coactivator 1-alpha, Nrf2: Nuclear factor erythroid 2-related factor 2, IL: interleukin, hs-CRP: high sensitivity *C*-reactive protein, TMAO: Trimethylamine *N*-oxide, NF-κB: nuclear factor kappa-light-chain-enhancer of activated B cells, TGF-β: transformin growth factor-β, ROS: reactive oxygen species, NLRP3: NOD-like receptor protein 3, sST2: soluble suppression of tumorigenesis-2 factor, TNF-α: tumor necrosis factor-α, LDL: low-density lipoprotein, sTWEAK: soluble tumor necrosis factor-like weak inducer of apoptosis. Regarding the effects of Diet/Nutrient on molecular pathways and on inflammation/metabolism, ↑ indicates increase, promotion or enhancement and ↓ indicates reduction/decrease/attenuation.

**Table 3:** RCTs and meta-analyses evaluating the impact of pharmacological agents on inflammatory biomarkers and cardiovascular events.

Study (Publication Year) (Reference)	Study Design	Patient Characteristics	Number of Patients	Agent	Outcome
Awad et al. (2022) [53]	Meta-analysis	Most patients with CAD, PAD, HTN, metabolic syndrome	3489	ACEIs/ARBs vs. placebo	Significant reduction in CRP, IL-6 and TNF-α with ACEIs, significant reduction in IL-6 with ARBs.
Wang et al. (2022) [54]	Meta-analysis	T2D	6261	SGLT2inh.vs placebo/antidiabetic drugs	Significant reduction in CRP with SGLT2inh.
He et al. (2023) [55]	Meta-analysis	Patients without history of CAD	26,521	Statins vs. placebo	Significant reduction in CRP with statins.
Oikonomou et al. (2019) [44]	Double-blind, placebo controlled, cross-over trial	Ischemic HF	31	Omega-3 PUFAs vs. placebo	Decrease in hs-CRP, sST2, increase in LVEF, GLS.
Sethuramalingam et al. (2023) [56]	Meta-analysis	CAD	N/A	Colchicine vs. placebo	Reduction in hs-CRP, reduction in composite endpoint of cardiovascular events and MI, no significant reduction in cardiovascular/all-cause mortality.
Ridker et al. (2017) [57]	RCT	Previous MI, hsCRP ≥ 2 mg/L	10,061	Canakinumab vs. placebo	Decrease in hs-CRP, cardiovascular events, no significant difference in cardiovascular mortality, higher incidence of fatal infection with canakinumab.
ATTACH trial (2003) [58]	RCT	HF (NYHA III-IV), mean LVEF: 24%	150	Infliximab vs. placebo	Reduction in CRP, IL-6 at 14 weeks, increase in all-cause mortality and HF hospitalization.
Ridker et al. (2019) [59]	RCT	MI/CAD	4789	Methotrexate vs. placebo	No significant reduction in CRP, IL-1β, IL-6 and cardiovascular events with methotrexate.

Abbreviations: CAD: coronary artery disease, PAD: peripheral artery disease, HTN: hypertension, ACEIs: Angiotensin-Converting Enzyme Inhibitors, ARBs: Angiotensin II Receptor Blockers, CRP: *C*-reactive protein, IL: interleukin, TNF-α: tumor necrosis factor-α, T2D: type 2 diabetes mellitus, SGLT2inh.: sodium-glucose cotransporter-2 inhibitors, HF: heart failure, PUFAs: Polyunsaturated Fatty Acids, hs-CRP: High-sensitivity *C*-reactive protein, sST2: soluble suppression of tumorigenesis-2 factor, LVEF: left ventricular ejection fraction, GLS: global longitudinal strain, N/A: not applicable, MI: myocardial infarction, RCT: randomized controlled trial, NYHA: New York Heart Association.

**Table 4:** Synergistic effects of dietary patterns/nutrients combined with pharmacological agents, molecular pathways-targets and potential cardiovascular benefits.

Nutrient–Therapy Combination	Molecular Pathways	Potential Cardiovascular Benefits
Mediterranean diet + statins	↑ AMPK, SIRT1, ↓ mTOR	↑ endothelial function and ventricular compliance
Metformin + low-carbohydrate diet	↑ AMPK	↑ exercise tolerance, ↓ oxidative stress
Polyphenol-rich diet + ACE inhibitors	↑ SIRT1, ↓ NF-κB	↓ fibrosis
Omega-3 PUFA + β-blockers	↑ PPARα, AMPK	↓ plasma triglycerides, ↓ arrhythmia burden

Abbreviations: AMPK: 5′ Adenosine monophosphate (AMP)-activated protein kinase, SIRT1: sirtuin 1, mTOR: mammalian target of rapamycin, NF-κB: nuclear factor kappa-light-chain-enhancer of activated B cells, PPARα: peroxisome proliferator-activated receptor alpha. Regarding the effects of Nutrient-Therapy Combination on molecular pathways and potential cardiovascular benefits, ↑ indicates increase, promotion or enhancement and ↓ indicates reduction/decrease/attenuation.

## Data Availability

Not applicable.

## Abbreviations

The following abbreviations are used in this manuscript:

HF	Heart failure
EF	ejection fraction
DII	Dietary Inflammatory Index
CRP	*C*-reactive protein
hs-CRP	high sensitivity *C*-reactive protein
IL	interleukin
SGLT2	sodium glucose transporter-2
TNF-α	tumor necrosis factor-α
RCTs	randomized controlled trials
ROS	reactive oxygen species
HFrEF	heart failure with reduced ejection fraction
HFmrEF	heart failure with mildly reduced ejection fraction
HFpEF	heart failure with preserved ejection fraction
CRT	cardiac resynchronization therapy
RAAS	renin–angiotensin–aldosterone system
TLR4	Toll-like receptor 4
NLRP3	NOD-like receptor protein 3
sST2	soluble suppression of tumorigenesis-2 factor
TMAO	Trimethylamine *N*-oxide
CKD	chronic kidney disease
NADPH	nicotinamide adenine dinucleotide phosphate
LDL	low-density lipoprotein
NO	nitric oxide
HIF-1α	hypoxia-inducible factor 1α
PRR	pattern-recognition receptor
ICAM-1	intercellular adhesion molecule
VCAM-1	vascular cell adhesion molecule
TNFR	TNF receptor
ECM	extracellular matrix
LPS	lipopolysaccharide
TGF-β	transforming growth factor-β
CCL2	C-C Motif Chemokine Ligand 2
MCP-1	Monocyte Chemoattractant Protein-1
MitoDAMP	damage-associated molecular pattern
NF-κB	nuclear factor kappa-light-chain-enhancer of activated B cells
NET	neutrophil extracellular trap
ACS	acute coronary syndrome
RAGE	Receptor for Advanced Glycation Endproducts
mPTP	mitochondrial permeability transition pore
NP	natriuretic peptide
BNP	B-type natriuretic peptide
NT-proBNP	*N*-terminal proBNP
NLR	neutrophil-to-leucocyte ratio
ADHF	acute decompensated heart failure
GDF-15	growth/differentiation factor 15
ATP	Adenosine Triphosphate
AMP	Adenosine Monophosphate
AMPK	5′ Adenosine Monophosphate-activated protein kinase
SIRT1/PGC1-α	sirtuin 1/peroxisome proliferator-activated receptor gamma coactivator 1-alpha
MAPK	mitogen-activated protein kinase (MAPK)
PPARα	peroxisome proliferator-activated receptor alpha
Nrf2	Nuclear factor erythroid 2-related factor 2
PAF	platelet-activating factor
Lp-PLA2	lipoprotein-associated phospholipase A2
miRNA	microRNAs
NAFLD	non-alcoholic fatty liver disease
ALT	Alanine Aminotransferase
AST	Aspartate Aminotransferase
HDL	high-density lipoprotein
CoQ10	coenzyme Q10
LVEF	left ventricular ejection fraction
sTWEAK	soluble tumor necrosis factor-like weak inducer of apoptosis
ACEI	Angiotensin-converting enzyme inhibitors
ARB	angiotensin receptor blockers
ARNI	angiotensin receptor neprilysin inhibitor
MRA	mineralocorticoid receptor antagonists
IFN-γ	interferon gamma
*n*-3 PUFA	*n*-3 polyunsaturated fatty acids
eGFR	estimated glomerular filtration rate
GLS	global longitudinal strain
NAD	nicotinamide adenine dinucleotide
CAD	coronary artery disease
PAD	peripheral artery disease
HTN	hypertension
T2D	type 2 diabetes mellitus
MI	myocardial infarction
NYHA	New York Heart Association
mTOR	mammalian target of rapamycin
6MWD	6 min walking distance
CMR	cardiac magnetic resonance
